# Competitive Trace Theory: A Role for the Hippocampus in Contextual Interference during Retrieval

**DOI:** 10.3389/fnbeh.2013.00107

**Published:** 2013-08-12

**Authors:** Michael A. Yassa, Zachariah M. Reagh

**Affiliations:** ^1^Department of Psychological and Brain Sciences, Johns Hopkins University, Baltimore, MD, USA

**Keywords:** systems consolidation, multiple trace theory, pattern separation, pattern completion, interference, episodic memory, semantic memory, competition

## Abstract

Much controversy exists regarding the role of the hippocampus in retrieval. The two dominant and competing accounts have been the Standard Model of Systems Consolidation (SMSC) and Multiple Trace Theory (MTT), which specifically make opposing predictions as to the necessity of the hippocampus for retrieval of remote memories. Under SMSC, memories eventually become independent of the hippocampus as they become more reliant on cortical connectivity, and thus the hippocampus is not required for retrieval of remote memories, only recent ones. MTT on the other hand claims that the hippocampus is always required no matter the age of the memory. We argue that this dissociation may be too simplistic, and a continuum model may be better suited to address the role of the hippocampus in retrieval of remote memories. Such a model is presented here with the main function of the hippocampus during retrieval being “recontextualization,” or the reconstruction of memory using overlapping traces. As memories get older, they are decontextualized due to competition among partially overlapping traces and become more semantic and reliant on neocortical storage. In this framework dubbed the Competitive Trace Theory (CTT), consolidation events that lead to the strengthening of memories enhance conceptual knowledge (semantic memory) at the expense of contextual details (episodic memory). As a result, remote memories are more likely to have a stronger semantic representation. At the same time, remote memories are also more likely to include illusory details. The CTT is a novel candidate model that may provide some resolution to the memory consolidation debate.

## Introduction

Much evidence points to the significant role of the hippocampus in the encoding of new declarative memories (Milner et al., 1998; Squire, 2009). This small region of the brain possesses a unique architecture that allows it to rapidly encode experiences while minimizing interference. Virtually every model of learning ascribes this important function to the hippocampus, especially in the context of declarative memory (in contrast to habit or other procedural learning). However, the role of the hippocampus in retrieval (especially episodic retrieval) is still subject to debate. While there is certainly a neural architecture in the hippocampus capable of such contextual retrieval, for example, a recurrent collateral network in CA3 capable of autoassociation (Marr, 1971), as well as an abundance of evidence across species demonstrating the involvement of the hippocampus in contextual retrieval tasks (Eldridge et al., 2000; Ryan et al., 2001; Yonelinas et al., 2002, 2005; Holdstock et al., 2004; Daselaar et al., 2006; Diana et al., 2007; Wiltgen et al., 2010; Goshen et al., 2011) there is still an active debate about whether the hippocampus is required for retrieval of *remote* episodic memories.

### Systems consolidation vs. multiple trace theory

At present, two major theories make predictions relevant to this debate. The first is the Standard Model of Systems Consolidation (SMSC: Squire and Alvarez, 1995), a widely influential view in the field. The SMSC holds that the initial memory trace is encoded both in the hippocampus and in the cortex, though the cortex is itself unable to initially support the memory. Rather, the hippocampus is critical in early encoding stages. As a function of time, replay, and retrieval, the hippocampus “teaches” the cortex the memory trace such that the associative connectivity between the individual elements of the cortical memory increase in strength over time. After the memory has been consolidated, the hippocampus is no longer required for retrieval. This is based on the large body of evidence that synapses change much more rapidly and dynamically in the hippocampus than they do in cortex (Frankland and Bontempi, 2005). These ideas were first proposed by Marr (1971) and further elaborated by the widely influential Complementary Learning Systems (CLS) model of McClelland et al. (1995), which emphasizes the role of hippocampal-neocortical interactions in the formation and consolidation of memory. Thus, the SMSC predicts that the hippocampus is not required for the retrieval of remote memories, only recent ones that have not yet been fully consolidated.

The competing theory, known as Multiple Trace Theory (MTT), was proposed by Nadel and Moscovitch (1997) as an alternative to the standard model. Unlike the SMSC, MTT proposed that the hippocampus has an important role in the retrieval of all episodic memories, including remote ones. Similar to the SMSC, MTT also proposed that memories are encoded in hippocampal-neocortical networks, but that each reactivation resulted in a different trace in the hippocampus. Hippocampal-bound traces are presumed to be contextual and rich in spatial and temporal details, while cortical-bound traces are presumed to be semantic and largely context-free. Thus, retrieval of remote semantic memories does not require the hippocampus, however, retrieval of remote episodic memories always does, irrespective of the age of the memory.

Thus, at the heart of the debate is the role of the hippocampus in the retrieval of remote episodic memories. In fact, that has been the only reliably testable prediction for either theory thus far, although as discussed below, support for even this single prediction proved tenuous at best. First, it is important to recognize that both models were proposed to explain amnesia data from human and animal studies, and namely the nature of the retrograde amnesia (RA) gradient observed. While numerous studies have observed that the RA gradient was temporally graded, in many cases, the RA gradient was flat, and in some cases the degree of RA gradient depended on the size of the lesion (reviewed in Frankland and Bontempi, 2005). In addition to lesion data, data from functional magnetic resonance imaging (fMRI) has been brought to bear on this debate. For example, Nadel and Moscovitch (1997) have shown that medial temporal fMRI activity was equally predictive of recent and remote memory retrieval. However, a major criticism of these studies is that the hippocampus is involved in incidental and automatic encoding during retrieval tasks, which may obscure retrieval-related activity (Buckner et al., 2001; Haist et al., 2001; Stark and Okado, 2003). A recent survey of the evidence based on amnesia studies in rodents with partial and full hippocampal damage provides overwhelming support for flat RA gradients, which argues against the SMSC (cf. Sutherland et al., 2010). Importantly, however, these data also argue against one prediction of MTT, which is that *partial* hippocampal damage will lead to a temporal RA gradient. Thus, neither model can adequately account for lesion data in animals.

Two particularly compelling pieces of data are worth discussing here to further illustrate the complexity of this debate. Scoville and Milner (1957) initially reported that patient H. M. had a case of temporally graded RA. In fact, much of the subsequent work on RA was based on this initial finding. Much later, however, as it became clearer that neuropsychological testing procedures were not as refined in that era and that episodic memory could not have been tested fully. Corkin (2002) later asserted that “H. M. was unable to supply an episodic memory of his mother or his father – he could not narrate even one event that occurred at a specific time and place.” She surmised that many of the remote memories H. M. was able to retrieve were indeed “semanticized.” In contrast, patient E. P., another case of profound amnesia studied by Squire and colleagues, was able to demonstrate highly detailed spatial remote memories (Stefanacci et al., 2000), arguing against the notion of a flat episodic RA gradient. This is further complicated by the inconsistency of results across studies of different amnesic patients with partial medial temporal lobe or hippocampal damage, and the lack of detailed neuroanatomical quantification in many cases. Thus, evidence from amnesia as to the RA gradient is not entirely conclusive, and provides only partial and sometimes conflicting support for either of the major theories discussed above.

A recent study by Goshen et al. (2011) used optogenetic techniques to demonstrate that hippocampal CA1 neuron activation was necessary for the retrieval of several week old (i.e., remote) memories, providing evidence against the SMSC. However, they also showed that longer inhibition (matching the timescale of the more typical pharmacological inhibition) abolished this dependence on the hippocampus, weakening the account provided by MTT. While Goshen and colleagues suggested that there is compensation via other structures such as the anterior cingulate cortex, the data can be taken to suggest both MTT and SMSC may both be at work and that perhaps each offers elements of the true nature of memory consolidation. This recent work motivates and underscores the value of alternate proposals that attempt to harmonize between the two models.

### The hippocampus as an index

Both accounts discussed above rely to an extent on the notion of hippocampal indexing. These ideas were initially presented by Teyler and DiScenna (1985), and were formally developed into the hippocampal memory indexing theory (Teyler and DiScenna, 1986). This theory has served as a critical component of our current understanding of hippocampal computations, and as such, it warrants discussion here (for review, see Teyler and Rudy, 2007).

The central aim of the hippocampal memory indexing theory is to explain the nature of hippocampal involvement in encoding and retrieving memory traces. Particularly, this was among the first attempts at explaining interactions between the hippocampus and neocortex during episodic memory computations. Though evidence had accumulated to underscore the importance of the hippocampus in many memory processes, two important realizations came to light. First, there appeared to be multiple neural networks capable of supporting memory (Sherry and Schacter, 1987). Second, the neocortex itself was found to be sufficient to support some aspects of memory (Squire et al., 1984). Tulving and Markowitsch (1998) went on to propose that episodic memory – that is, memory rich in associated contextual details – is especially dependent on the hippocampus. Indexing theory describes the involvement and ultimate fate of these contextual details.

According to this theory, when a memory trace is encoded, inputs from cortical sensory regions activate a relatively small population of hippocampal synapses. The hippocampus in turn activates a network of neocortical regions, and as the memory is consolidated, the connections between the hippocampus and neocortex are strengthened. Laying down hippocampal-neocortical connections in this manner creates a physical instantiation of the memory trace. Importantly, the hippocampus here plays a pivotal role in memory retrieval. Activation of a small subset of neocortical regions, part of a larger pattern comprising a consolidated memory trace, can signal the hippocampus to re-instantiate the full pattern despite partial or degraded input. In short, this provides an account for how certain aspects or contextual details of an event can lead to recall of other related details.

It deserves further emphasis that under this interpretation, the hippocampus does not store details about an event *per se*, but as the name of the theory implies, rather acts as an index. That is, as was described in Teyler and DiScenna’s theory, the hippocampus is proposed to serve in coupling the activity of neocortical regions such that patterns of activity can induce recall of a given memory trace. To make this proposition as clear as possible, let us consider another description. If information is stored across the neocortex, we might imagine it as a library. Memories, much like library books, are often added, removed, or replaced. When reconstructing an experience, one may need to access information residing in different wings of the library. This is where the hippocampus, our trusty librarian, comes in. While it has not stored the wealth of knowledge contained in the library in a way that it can readily reproduce, it can point to the correct locations where this knowledge can be retrieved.

## Competitive Trace Theory

We propose an alternative to the current theories of recent and remote memory that combines elements of SMSC and MTT largely within the framework of indexing theory. The account, which we will refer to as the competitive trace theory (CTT), is an integrated theory that attempts to explain phenomenological distinctions such as episodic vs. semantic, using neurocomputational proposals based on interference and associations.

### Consolidation and decontextualization

First, we will start with some operational definitions. The words “episodic” and “semantic” have been used abundantly in the memory literature to refer to memories that are rich in contextual detail and memories that are devoid of such details, respectively. However, there is an additional important distinction that should be considered here. That is the accuracy of such memory, which is often uncorrelated with the success of recollection (Gallo et al., 2001; Roediger et al., 2004; Kensinger and Schacter, 2007; Stahl and Klauer, 2008; Kim and Yassa, 2013). Thus, in the CTT framework, the word “episodic” will only be used to describe the phenomenological experience of contextual recollection and not in reference to the accuracy of the memory. Inherent in this assignment is the strong claim that these labels (“recollection” and “episodic”) are only helpful insofar as they describe the experience and not describe the memory representation itself, which is far more dynamic and often contains illusory details.

The word “semantic,” on the other hand, will be used to refer to the accurate knowledge that builds up over time and with much repetition. The use of these terms will become more defined as we describe the central tenets of the model, and we will maintain that their use is only helpful in relative terms and not absolutes (i.e., one memory can be *more episodic than* another, but should not be labeled as “episodic” absent a frame of reference). For now, it is important to bear in mind three crucial assumptions of CTT: (1) memories are most episodic *and* veridical at the moment they are first encoded, (2) with every subsequent reactivation, the memory can become less episodic, and accurate details can be replaced with illusory details, and (3) central features of experiences become simultaneously consolidated and decontextualized (lose associated details) over time.

How does this occur? We suggest that when a memory is reactivated by an internal or external cue, the hippocampus acts to re-instantiate the neural signature of the original memory trace. In doing so, the hippocampus effectively recombines the elements of the original memory trace. Critically, the central features of that memory trace are reactivated. However, unlike prior theories of episodic memory retrieval, we propose that this process potentially adds or subtracts individual contextual features. Given the reactivation of the central features of the memory trace, the new memory significantly overlaps with the original. However, some of the features are non-overlapping, which leads to a slightly altered version of the memory. This altered memory is now capable of being stored as a new memory trace and undergoes the same storage process as the original memory. This in some ways is reminiscent of MTT, but with several important distinctions. According to our proposal, these memories are not stored in parallel, but rather compete for representation in the neocortex. Also, MTT hypothesizes that the memory traces themselves are stored in the hippocampus and not in the neocortex (this is the logic behind the “larger lesions knock out older memories” effect (Nadel and Moscovitch, 1998). Neocortical traces, according to MTT, are overlapping only insofar as the encoding and retrieval contexts are overlapping, which allows for contextual retrieval driven by hippocampal or neocortical traces. CTT, on the other hand, hypothesizes that the hippocampus itself is not the site of trace storage but rather it links the individual components of a neocortical memory together such that it can be retrieved later by the hippocampus or by the neocortex directly. Furthermore, neocortical traces themselves become devoid of context with increasing reactivations.

Two distinct phenomena can occur here: consolidation and decontextualization. First, overlapping features in the memories should not compete for representation and thus are strengthened (i.e., consolidated) in a Hebbian fashion. As a result of repeated activations, these overlapping features have a higher likelihood of being retrieved with high fidelity. The increase in associative connectivity over time allows these personal semantic components of the memory to become hippocampus-independent. That is, the overlapping neocortical components of such a memory trace have become strengthened to the extent that the hippocampus is no longer necessary to couple their activity. Second, the non-overlapping features should compete with one another resulting in mutual inhibition in an anti-Hebbian fashion, and a reduced likelihood of any of such features being retrieved. In other words, memories become decontextualized.

**Figure 1:**
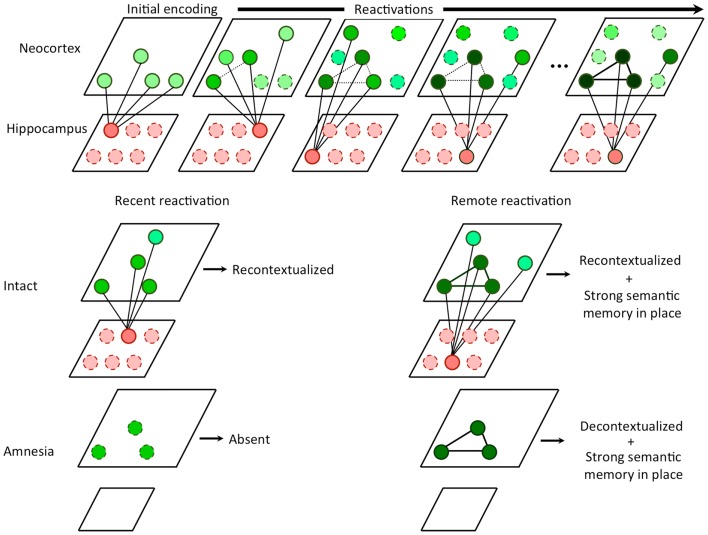
**Hippocampal competitive trace theory**. Every time a memory is reactivated, the hippocampus encodes a partially overlapping trace that serves to compete with other similar traces from other reactivations in the neocortex. In the hippocampus, traces are non-overlapping due to pattern separation. In the neocortex, the overlapping features are strengthened (i.e., consolidated) while the non-overlapping features become decontextualized. During recent memory reactivation, an intact hippocampus is able to recontextualizes the memory, storing an additional trace. In amnesia, the recent memory cannot be retrieved in the absence of the hippocampus, since no features have yet been consolidated. During remote memory reactivation, an intact hippocampus continues to recontextualizes the memory, however, a strong consolidated semantic memory is in place due to a number of prior reactivations. In amnesia, the remote memory can be retrieved at least in part based on the semantic memory. Absent the hippocampus, recontextualization can no longer occur and additionally, the retrieval experience may be less contextual than that of an intact subject.

It follows from the above that retrieval of remote memories appears episodic and contextual because of hippocampal reconstruction and re-encoding, rather than a reactivation of a veridical representation. Without the presence of the hippocampus during retrieval (as in amnesia), the only retrievable memory is the high fidelity semantic representation in the neocortex (see Figure 1). These highly semanticized memories, having been consolidated and reconsolidated, are likely to feature a core set of important facts but little contextual depth. Thus, CTT can be viewed as a harmonization of SMSC and MTT in which consolidation and hippocampal independence occurs for semantic components of experiences via a multiple trace mechanism, provided that the non-overlapping portions of these traces compete for representation.

The CTT model asserts that recent episodic memories and remote episodic memories, although they share phenomenological features such as the sense of recollection or mental time travel (Suddendorf and Corballis, 1997), possess underlying representations that could not be more different. A recent memory has no semantic components as those take time and repeated instances of remembering to build, but it is rich in accurate contextual detail. A remote memory, on the other hand, has a strong semantic component as a result of repeated retrieval events, but also contains degraded contextual information, or reconstructed contextual details that are often inaccurate.

Given these assumptions, we can redefine systems consolidation as the selective strengthening of the core content of the memory in neocortical circuits via hippocampal-neocortical interactions, coupled with a selective weakening of irrelevant and highly variable contextual details associated with each reactivation of the memory. It is important to note that the second condition of consolidation is most directly observable in hippocampal amnesia as the presence of the hippocampus in the intact brain gives the illusion of intact contextual detail, while in fact this experience is the direct result of mnemonic reconstruction and retrieval of illusory contextual details (Figure 1).

This raises the question of why the hippocampus continues to manufacture these illusory recollections, while a perfectly intact semantic memory is accessible in the neocortex. There are several potential answers. First, it is likely that contextual recollection, despite its inaccuracy, facilitates social interactions, and the sharing of experiences for the purpose of social bond formation. Second, reconstructing such details, which become influenced by cultural and other personal biases, may serve the important adaptive role of creating narratives that can influence others’ behavior. Imagine, for example, how compelling reading someone’s autobiography can be. Of course, another alternative is that there is no evolutionary advantage to recollection aside from facilitating the competition that is used to abstract memories so that massive amounts of information can be stored. In this sense, it may better facilitate learning and future adaptive behavior to incorporate illusory details into a memory than to simply forego details altogether, and the recollective experience may be nothing more than an epiphenomenon of an otherwise adaptive system.

### The recontextualization continuum

**Figure 2:**
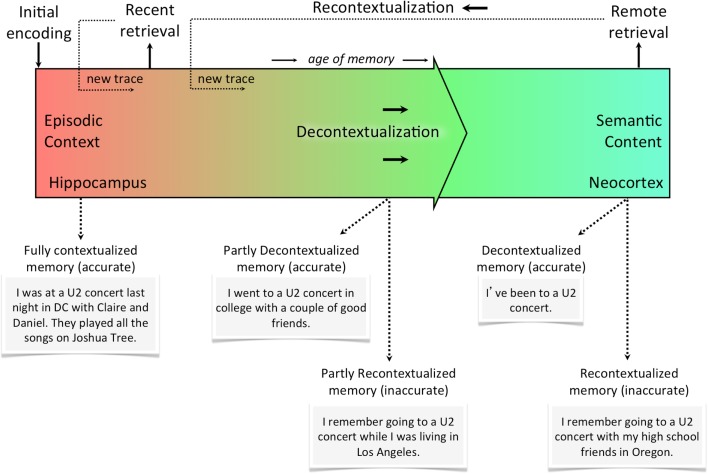
**The recontextualization continuum**. Memories are most rich in episodic context the moment they are first encoded. With every subsequent reactivation (whether it’s internally or externally cued), the memory becomes less episodic and more semantic (i.e., decontextualized). Every time a memory is retrieved, however, it can be recontextualized or re-encoded as a new trace with some overlapping features and some new ones. This means memories recalled from the distant past could either be decontextualized and accurate or recontextualized and inaccurate. Of course, neither accuracy or contextualization is a categorical assignment for the memory, but rather each memory can be described in terms of the accuracy of its details and the degree to which they are contextual (i.e., episodic).

Figure 2 illustrates the episodic-semantic memory continuum (purely based on the retrieval experience, not taking into consideration the accuracy of memory). Given the slow cortical dynamics responsible for consolidation, this relationship is best represented as a continuum of decontextualization/recontextualization. In other words, the axis of this continuum is the degree of contextual detail (a function of reactivation events), which can be formally quantified. The hippocampus, at the very left of the continuum, is a context-encoding, associative device that simultaneously strengthens some components of the memory and distorts others. The neocortex, at the very right of the continuum, is the final storage site of semantic memories that have been consolidated using slow cortical dynamics and trace interference over time. At the neocortical stage, recurrent and overlapping details have become relatively crystallized as the memory trace has been reconsolidated, but non-overlapping details are more transient and may be unique to a given recollective experience. That is, retrieval at any point in time results in a new trace that is stored as a slightly alternate version of the original memory. Retrieval at different points in this continuum is shown using examples. Altering contextual details may involve a large distortion (e.g., misremembering the city in which something occurred) or a very small one (e.g., misremembering which side of the sofa you were sitting on). Importantly, however, any deviation from the original representation is likely to correspond to a deviation from the initial neural representation of that memory trace. Even if a given retrieval event produces a memory trace that highly overlaps with the original memory, any amount of difference may be sufficient to induce competition.

**Figure 3:**
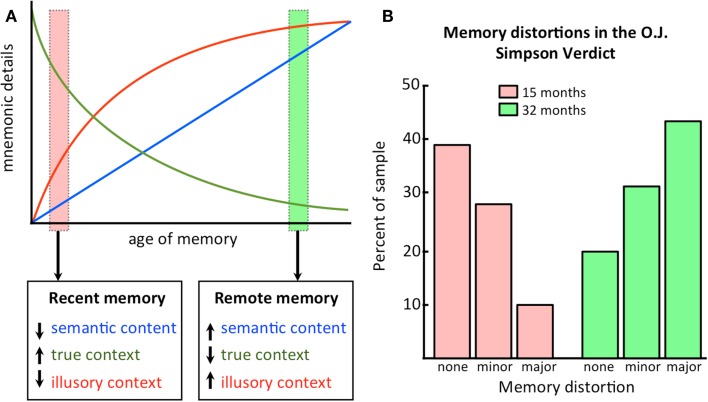
**Illusory details build up as memories get older**. **(A)** A conceptual representation of how information content changes as a function of the age of the memory. Semantic content increases with consolidation, while true contextual details are replaced by illusory details; **(B)** An empirical demonstration of how illusory details increase as a function of time. See text for discussion. Data plotted based on values from Schmolck et al. (2000).

Figure 3A is another demonstration of the change in contextual details over time that is predicted by CTT. Recent memories are high in accurate details, low in semantic content (which has not yet been consolidated), and low in inaccurate details, as the hippocampus has not yet had an opportunity to distort the memory owing to only a few replay/reactivation events. Remote memories, on the other hand, are low in accurate details, high in semantic content (which is now consolidated in the neocortex), and high in inaccurate details, as the hippocampus has had ample opportunity to distort the memory across numerous replay/reactivation events. The decline in accuracy of the memory follows the typical forgetting curve of Ebbinghaus (1885).

The increase in semantic strength is assumed to be monotonic and linear, although it is quite possible that it follows the same curvilinear pattern. Replay studies repeatedly demonstrate reactivation events in the short term (minutes, hours), with very few on the order of days (reviewed in Sutherland et al., 2010). Thus it is possible that most events leading to consolidation occur in the short term and the memory asymptotes quickly. However, in the absence of robust data for remote replay (exceeding several days), the exact pattern is difficult to infer. Importantly, CTT only makes assumptions about the slopes of these curves relative to one another and not about the exact shape of any particular curve.

To summarize, below are the central tenets of CTT:

1. Every time a memory is reactivated, the hippocampus recontextualizes the memory by re-encoding a similar but not identical memory trace that is stored using associative connectivity between the hippocampus and neocortex.
2. Memories are decontextualized over time by competitive interference among these similar but not identical multiple memory traces. This simultaneously leads to consolidation of semantic memory in the neocortex and loss of episodic details.
3. Veridical episodic details are only available in very recent memories. As memories get older, these details are replaced by recontextualized details stored by the hippocampus that give rise to illusory memories that become more prevalent as the age of the memory increases. In other words, recent episodic and remote episodic memories vary in accuracy of details and strength of semantic content.

## Harmonizing CTT with Pre-Existing Ideas

As we previously mentioned, CTT borrows elements from many existing theories and models. It is largely consistent with indexing theory (Teyler and DiScenna, 1986) and stresses the role of the hippocampus in encoding and binding the initial memory traces, and acting as an index during retrieval. Much like the CLS (McClelland et al., 1995) framework, CTT also assumes that the reactivation of hippocampal-neocortical traces strengthens the cortico-cortical traces leading to consolidation of memories. On the other hand, CTT also assumes that each reactivation of the memory results in a new trace and not just the reactivation of the old trace, which is consistent with MTT. Also consistent with the MTT proposal is the notion that the hippocampus is involved in the “reconstruction” rather than the “retrieval” of the memory. Nadel and Moscovitch (1998) also propose that reactivation of neocortical traces strengthens the links among multiple traces, which is the basis for building knowledge. This is further in agreement with CTT, however, we also suggest that the non-overlapping components of the traces compete with one another resulting in decontextualization in addition to consolidation.

We propose that the new hippocampal-neocortical traces formed are always partially but not completely overlapping with the original trace, resulting in competition for representation in the neocortex (this competition does not occur in the hippocampus due to pattern separation mechanisms discussed in detail below), leading to a selective strengthening of semantic information and weakening of contextual information. Thus, under CTT, the role of the hippocampus during retrieval is hypothesized to be the recontextualization of memories during retrieval to generate new competing traces. On the face of it, this may seem to be counterproductive at the level of the neocortex. As we will discuss below, we believe that this arises as a function of pattern separation computations necessary for episodic encoding. However, inducing competition may have a particular benefit in maintaining and retrieving memory traces. Namely, degradation of competing elements of a memory trace ensures that the important central features of that memory are not only preserved, but also strengthened.

The role of the hippocampus in recontextualization is also closely related to its role in mental imagery and imagining the future. Hassabis et al. (2007) demonstrated that this ability is impaired in hippocampal amnesic patients. They surmised that the hippocampus may contribute to the creation of new experiences by allowing disparate elements of prior memories to be bound in a spatial context. Addis and Schacter (2011) further extend this in a recent review of patient and neuroimaging findings to suggest that the hippocampus is also necessary for imagining the future and “episodic simulation.” These roles in imagery are consistent with the notion of hippocampal recontextualization that we propose herein.

Our view is also largely consistent with the Distributed Reinstatement Theory of Sutherland et al. (2010) in which it is the frequency of replay/re-encoding episodes rather than the passage of time that leads to memories becoming independent of the hippocampus. Indeed, the central tenet of CTT is that reactivation events occurring as the age of the memory increases are the critical event in consolidation. While “age of memory” is plotted along the abscissa in the illustrations, it is used merely as a proxy that makes measurement feasible. Quantifying the number of reactivations, which is likely non-linear, is much less feasible. While one can pit these two alternatives (age of memory vs. number of reactivations) against each other in an experimental setup, only cued, not spontaneous, reactivation events can be assessed easily.

One potential possibility using advanced optogenetics techniques is to allow the hippocampus to engage in initial learning which could label the neurons involved using an immediate-early gene (e.g., Liu et al., 2012), then quantify reactivation events occurring within the labeled population only. Better yet, by silencing these neurons during specific time epochs, only circumscribed reactivations could be allowed. Thus, the effect of the passage of time vs. number of reactivations can be assessed directly.

The notion of competition for representation is not unique to memory by any means, and in fact seems to be a general principle of cortical operation. For example, it generally believed that in order for objects in the visual field to capture attention and be subjected to further neural processing they compete with one another for representation. Bottom-up and top-down influences can bias this competition by assigning priority to certain features or items but not others (Desimone and Duncan, 1995). A similar argument has been extended to the role of arousal in increasing the contrast between important and unimportant details in mnemonic representation (Mather and Sutherland, 2011). Thus, competition among memory *traces* (in contrast to competition among memory *systems*, which is widely accepted) is not an implausible idea, and in fact could be supported by very similar mechanisms to competition in other domains.

We further propose that the mechanisms involved in this competition arise as a result of hippocampal-neocortical dynamics and are particularly dependent on hippocampal processing. This harmonizes the model with the hippocampus’s role in minimizing interference (i.e., pattern separation), a central tenet of the CLS. Below, we discuss this function in detail and suggest a potential mechanism by which it can facilitate cortical interference whilst minimizing hippocampal interference.

## Contributions of Pattern Separation and Pattern Completion

The hippocampus is capable of supporting rapid encoding of unique experiences by orthogonalizing incoming inputs such that interference is minimized, a function termed pattern separation, which is typically ascribed to the hippocampal dentate gyrus (DG) (Marr, 1971; Treves and Rolls, 1994; McClelland et al., 1995; O’Reilly and Norman, 2002; Norman and O’Reilly, 2003; Yassa and Stark, 2011). The hippocampus also has a well-recognized role in the formation of arbitrary associations using its recurrent collateral network in the CA3 subregion, a function termed pattern completion (Rolls, 2007). Recent evidence from animals (Nakazawa et al., 2003; Guzowski et al., 2004; Lee et al., 2004; Leutgeb et al., 2004, 2005, 2007; Vazdarjanova and Guzowski, 2004; Gold and Kesner, 2005; Kesner, 2007; McHugh et al., 2007) and humans (Bakker et al., 2008; Lacy et al., 2011) has provided strong support for the involvement of the hippocampus in these two important mnemonic computations. Though there is still some debate as to the precise nature of episodic memory, most in the field regard its core components as consisting of autobiographical details such as what occurred in addition to where and when. The capacity to orthogonalize overlapping input to create distinct memory traces, or to reinstate a particular memory trace based on partial or degraded input, are critical for encoding and remembering such details (Norman and O’Reilly, 2003; Norman, 2010). We suggest that pattern separation and pattern completion, provided they occur across different dimensions including space and time, are together necessary and sufficient to give rise to our episodic memory system with all of its richness, associativity, and flexibility (Yassa and Stark, 2011).

Given this deeper understanding of episodic memory mechanisms, it is important to discuss how the proposed CTT framework fits with these computations. First, let us make the case for pattern completion. This ability requires the reactivation of a previously stored representation when presented with a partial or a degraded cue. It is hypothesized to be a specific function of the CA3 region of the hippocampus due to recurrent collateral connectivity, which forms an autoassociative network and its innervation from the neocortex via the perforant path. This rapid retrieval is also balanced against new encoding in the CA3 region, which is subject to strong input by mossy fiber innervation from the DG granule cells (a pattern separation signal) (Treves and Rolls, 1994). Thus, the CA3 region regulates the dynamic balance between pattern separation and pattern completion at least in the spatial domain. A similar role may exist for the CA1 region in temporal pattern separation and completion, though there is less existing data on this phenomenon (see Hunsaker and Kesner, 2013 for a comprehensive recent review).

We suggest that pattern completion in the hippocampus reactivates the neocortical trace and leads to a strengthening of the overlapping trace over time. A pattern completion mechanism is necessary for CTT and could in theory underlie the ability to strengthen representations over time in a Hebbian fashion (using a slow cortical dynamic). The decay in non-overlapping features of the memory due to competitive interference is likewise presumed to occur in anti-Hebbian fashion.

Next, we turn to pattern separation. An important tenet of CTT is that every time a memory is reactivated, re-encoding of the trace occurs. This re-encoding contains some of the reactivated features (the attractor state), in addition to some unique associations that attempt to orthogonalize, though incompletely, the current representation from past memories. According to CTT, pattern separation in the hippocampus (in particular, the DG) is the most important factor in re-encoding a slightly different version of the experience (i.e. recontextualization), which causes the subsequent competition among overlapping traces in the neocortex. This can be viewed as a side effect of an otherwise very adaptive process, which acts to minimize interference in the initial storage of information, but *leads to competitive interference* in the cortex over time as previous memories are reactivated. This is a much more dynamic view of pattern separation and attempts to examine its long-term not just short term effects. It is important to note that this type of interference is unlike the catastrophic interference that would occur if sequential learning occurred too rapidly. Cortical interference is much slower and thus is much more stable in terms of network dynamics. Given the above, CTT is not only consistent with the pattern separation/completion framework but in fact relies on these computations to formulate its predictions.

## Harmonizing CTT with Existing Data

Competitive trace theory is generally consistent with, and offers explanations for, much of the existing episodic memory literature across species. While discussing every bit of evidence in the field is beyond the scope of this article, we present the case here using specific representative examples from behavioral studies of false memory and neuropsychological studies of amnesia in animals and humans.

### Behavioral Studies of False Memory

To see the extent to which memory is non-veridical and is subject to constant updating, one need not look any further than the pioneering work of Bartlett (1932). Using the method of serial reproductions with material ranging from abstract drawings to stories such as “War of the Ghosts,” Bartlett illustrated beautifully how memory can be altered every time it is retrieved. While this insightful work taught us about the impact of social bias on remembering, it also demonstrated unequivocally that memory is not veridical and is subject to constant change and reconstruction. Since Bartlett, research in false memory has enjoyed a rich tradition. Loftus (2005) has been investigating how humans adopt misinformation for over 30 years providing much of what we know about how false memories can be formed and how they can be extraordinarily rich in complexity and detail. False recall is also easily demonstrated by memory tasks such as the Deese–Roediger–McDermott (DRM) paradigm (Deese, 1959; Roediger and McDermott, 1995), and mnemonic discrimination tasks with similar lures (Yassa et al., 2011). Schacter discusses these memory “sins” as features of an adaptive memory system (Schacter, 1999). The frequency and abundance of these phenomena are consistent with the premise of recontextualization in CTT and suggest that reactivations lead to reconstructions of and updates to the initial memory.

Aside from the mere existence of false memory, for which there is extensive evidence within our field, CTT further proposes that the probability by which false memories are created are increased with repeated reactivations. Since reactivations are difficult to assess directly in humans unless they are induced using a cueing procedure, one can use the age of the memory as a proxy. We predict that the more time passing since initial encoding would be associated with increased tendency for false memories or distortions. In other words, the number of veridical details reported would decline with the age of the memory, while the number of illusory details would increase. There are many demonstrations of this effect, but we will discuss just two examples here.

The first example comes from a study by Schmolck et al. (2000) where college students were asked to recall the circumstances surrounding hearing about the verdict in the O. J. Simpson double murder trial after 3 days and were re-tested on their memories 15 or 32 months later. They found that as a function of a longer retention interval, the frequency of memory distortions increased (at 32 months, more than 40% of the recollections contained major distortions and only 29% were highly accurate). These results are shown in Figure 3B.

The second example comes from flashbulb memories. Brown and Kulik first described the flashbulb memory in 1977 as a vividly detailed memory of the circumstances surrounding an important emotional event, such as the assassination of John F. Kennedy (Brown and Kulik, 1977). Neisser (1982) wrote of his own flashbulb memory of the Japanese attack on Pearl Harbor on Sunday, December 7th, 1941. He recalled that he was listening to a baseball game on the radio. Many years later, it occurred to him that no baseball games are played in December (it was later suggested that it was actually a football game). It is now well accepted that although flashbulb memories are high in vividness, accuracy in many cases is low. A more recent study by Talarico and Rubin (2003) suggested that confidence in flashbulb memories increases while accuracy decreases over time. The investigators tested college students on their memory of first hearing about the September 11th terrorist attacks the day after the events occurred. Repeat testing occurred at 1, 6, or 32 weeks later. They found that the decline in accuracy for flashbulb memories was no different than everyday memories, however ratings of vividness and confidence did not decline for flashbulb memories. This report is also consistent with CTT’s predictions, as the inclusion of fictitious details over time may give the illusion of accuracy and thus, recollection confidence (i.e., metamemory) remains high.

### Neuropsychological studies of human amnesia

While it is commonly agreed upon that cases of human amnesia suggest that remote retrograde memory is relatively intact, there is less agreement about whether the intact memories are rich enough in contextual detail to be deemed episodic or whether the recalled memories are more semantic in nature. SMSC asserts that those memories are truly episodic as they have become consolidated and become independent of the hippocampus long before hippocampal damage occurred. MTT, on the other hand, asserts that these memories are not entirely episodic because the hippocampus continues to be required for remote episodic memory recall.

As previously mentioned, Corkin characterized H. M.’s memories as “semanticized” or lacking in episodic detail, which is consistent with MTT. Other amnesia cases have demonstrated similar deficits in remote memories (Hirano and Noguchi, 1998; Moscovitch et al., 2000; Cipolotti et al., 2001). However, work by Squire and colleagues has strongly suggested that with more detailed neuropsychological investigations, the quality of retrieved remote memories in amnesia is similar to controls (Bayley et al., 2003; Kirwan et al., 2008). Squire and colleagues argue that the impairment in remote memory found in some cases of amnesia is secondary to non-MTL damage. In the absence of detailed neuroanatomical quantification, it is difficult to know whether this is truly the case. Another potential confound is the absence of corroboration to ensure the veracity of these memories (e.g., informant or diaries) in most if not all cases. Thus, it is not known whether the details retrieved are accurate or fictitious.

While the literature on remote memory in human amnesia is subject to much debate with respect to the episodic nature of such memories, CTT’s predictions have much to do with the veracity of these memories. Similar to MTT, it hypothesizes that the hippocampus continues to be important for remote memories, but for entirely different reasons. During recall of remote memories, the hippocampus recontextualizes or updates the memory. In its absence, a strong personal semantic memory is available in the cortex and can be accessed directly. It is important to note here that MTT proposes that retrieval is dependent on the hippocampus because the hippocampus is required to reconstruct the memory of the episode within a spatial scaffold (Nadel and Moscovitch, 1998), thus a non-hippocampal memory would lack spatial context. CTT, on the other hand, proposes that any context (spatial or otherwise) can additionally be consolidated and strengthened to become independent of the hippocampus, as long as it is overlapping and not interfering with prior exposures. The critical difference between the two models is the explicit role assigned for overlap and interference in CTT.

Whether this personal semantic memory has associated contextual detail is not a categorical distinction but rather depends on the position of this memory on the contextualization continuum previously discussed. Thus, some memories may have more contextual details than others. The counterintuitive prediction of CTT here, however, is that amnesic patients will have remote memories that are more accurate than healthy controls, since the absence of a hippocampus prevents the recontextualization and reconstruction of those memories. While the data supporting this account are only circumstantial, autobiographical memory reports from patients like E. P. do suggest that autobiographical memories were less likely to be embellished or changed despite repeated recall in amnesia (Bayley et al., 2003).

Overall, the data from human amnesia cannot be used as strong support for CTT or any other model for recent vs. remote memory, given the disagreements about the (1) quality (e.g., richness, vividness, etc.) of the memories retrieved, (2) quantity of the memories retrieved, (3) accuracy of the memories retrieved, and (4) neuroanatomical characterization of medial temporal lobe damage. It is our hope, however, that the additional predictions afforded by CTT provide a platform for future studies with amnesic patients that may support or refute some of these basic ideas.

### Rodent models of retrograde amnesia

Most investigations of RA in the rodent hippocampus have been conducted using contextual fear conditioning. While several early examinations of recent vs. remote memories reported a temporal RA gradient (Kim and Fanselow, 1992; Maren et al., 1997; Anagnostaras et al., 1999), other studies have reported flat RA gradients (Lehmann et al., 2007; Sutherland et al., 2008). Investigations of RA in hippocampus lesioned rats in spatial navigation tasks have also reported generally flat RA gradients (Sutherland et al., 2001; Clark et al., 2005a,b; Martin et al., 2005). CTT predicts that the extent to which the hippocampus is critical for remote retrieval (i.e., whether there is a temporal or flat RA gradient) depends on (1) how much reactivation has occurred since the initial learning and (2) the nature of the retrieval task and whether it requires hippocampal recontextualization. The latter point is one deserving of further analysis. The ability of the hippocampus to engage in recontextualization should be a function of all of the other experiences it has encoded (these are the sources of interfering traces that can compete with the memory with each reactivation), thus factors such as rearing in rich vs. impoverished environments can significantly influence the results.

This is evidenced by phenomena such as immediate shock deficit (ISD: Fanselow, 1986) and the context pre-exposure effect (Fanselow, 1990), where a critical role of the hippocampus in learning about the environment in contextual fear conditioning is demonstrated. It is likely that the parameters of these phenomena (e.g., latency required for ISD or pre-exposure) are dependent on the animal’s prior history. Most studies with rodents use individually housed rats in impoverished conditions, which results in the hippocampus operating under suboptimal conditions. We suggest that the hippocampus’ ability to facilitate cortical interference by encoding recontextualized versions of the memories will depend on this prior history. In light of this, a re-examination of lesion studies in rodents and future studies using animals reared in enriched environments are required to fully test the predictions of CTT.

Several studies have shown that hippocampal learning can compete with learning in non-hippocampal systems (Maren et al., 1997; Frankland et al., 1998; Driscoll et al., 2005; Lehmann et al., 2006; Sutherland et al., 2006; Wiltgen et al., 2006), suggesting that different memory traces do compete for representational resources in the brain. In a recent demonstration, Sutherland and colleagues (Sparks et al., 2011) showed that a non-hippocampally acquired contextual fear memory (learned while hippocampus was temporarily inactivated) was susceptible to interference or competition from the hippocampus when it was subsequently reactivated. These findings are directly predicted by CTT and further demonstrate the impact of competition among hippocampal-neocortical memory traces. These data also offer an alternative account to demonstrations of anterograde amnesia following pre-training hippocampal inactivation (e.g., Bast et al., 2001). While it is possible that the inactivation prevented the animals from learning the task, another possibility is that the reactivation of the hippocampus after learning disrupted performance on test due to competition with memory traces formed outside the hippocampus.

The above data suggest that there is indeed interference and competition between hippocampal and neocortical memories. The CTT formalizes this competition and describes a potential mechanism (via hippocampal pattern separation) by which it can occur.

### Memory updating and reconsolidation

It has been long known that retrieved memories are labile and can be disrupted. For example, Donald Lewis’s seminal experiments in 1968 demonstrated that reactivated memories can be disrupted by electroconvulsive shock (Misanin et al., 1968). Based on this work, Lewis proposed that reactivating a memory brings it into an active state that is vulnerable to disruption by external agents (Lewis, 1979). In 2000, Nader et al. (2000a) demonstrated that a protein synthesis inhibitor (anisomycin) resulted in the disruption of a reactivated fear memory. The authors proposed a mechanism for this disruption they termed “reconsolidation,” which essentially posits that reactivation results into two distinct events: an unbinding of the synapses representing the memory and a concurrent second round of protein synthesis to re-instantiate the memory. Protein synthesis inhibitors, they surmised, blocked the second event, thus disrupting the memory permanently (Nader et al., 2000b). Although initial reports were inconsistent across laboratories [e.g., memory loss was not always permanent (see Power et al., 2006)] and the widespread effects of protein synthesis inhibitors were seen as potential confounds (Rudy et al., 2006), more recent data has partly supported the notion that reconsolidation may occur under some conditions and may be at least one way in which memory updating can occur (Besnard et al., 2012).

Conceptually, CTT is consistent with both Lewis’s *Active Trace Theory* and reconsolidation theory in that it puts great emphasis on memory reactivation as the critical event by which a memory can be updated. We also similarly suggest that intervening with the memory trace during retrieval can disrupt it. However, the exact mechanisms diverge. CTT’s account is based on interference among competing memory traces and not a synaptic “resetting” *per se*. The extent to which spontaneous recovery can be observed (as in extinction procedures) will depend on the extent of the interference among the competing traces. If the competition results in suppression of much of the original memory, spontaneous recovery may not be observed.

More directly relevant to CTT, Monfils et al. (2009) recently suggested that preceding an extinction procedure with a single reactivation trial can disrupt fear memory. The same procedure has recently been applied to disrupt fear memories in humans (Schiller et al., 2010) and decrease cue-induced craving in human heroin users (Xue et al., 2012). While the results of this type of memory disruption have been interpreted in terms of reconsolidation, they do not necessarily speak to the underlying mechanism. We argue that results from retrieval-extinction procedures are more consistent with the CTT account, where retrieval is associated with a re-encoding of the memory, which only partially overlaps with the original memory and can compete with it for storage. In the retrieval-extinction procedure this process is greatly accelerated by providing the competing memory directly in the extinction trials.

It is likely that this update process happens all the time, however, there is typically no systematic attempt to extinguish behavior with competing memories in every day circumstances. This results in a subtle update that removes some features of the memory and adds others. In an experimental setting, however, where a competing memory is explicitly encoded to extinguish the fear behavior, it is much easier to observe a complete or near-complete ablation of the memory. Episodic memories in humans are also likely much richer than fear memories in animals thus a complete ablation would be more difficult to instantiate as the competition needs to occur repeatedly and over an extended period of time.

## Making New Predictions Based on CTT

The CTT framework represents a plausible mechanism by which hippocampal-neocortical traces are established and updated. It makes a set of empirical predictions that can be tested directly in animals or humans. We highlight some of these predictions here, in hopes that they will be instigate such research in the future to attempt to support or refute CTT’s core premises.

- The first prediction is the remote episodic memories should be less accurate than recent episodic memories. We discussed some evidence for this in studies of false memory as well as human amnesia, however the prediction needs to be tested more directly using veridical records of information. This prediction may also be tested using an animal model where the accuracy of the memory can be tested using a discrimination/generalization procedure. For example, Wiltgen and Silva (2007) provide supporting evidence for this by demonstrating that context generalization increases as a function of time, consistent with our proposal that more remote memories are less context-specific.
- The second prediction is that amnesia patients as well as rodents with hippocampal lesions should have more accurate remote semantic memories compared to healthy controls. This follows from the premise that having a hippocampus has the capacity to distort the memory every time it is recalled. Removing this brain region should also remove the capacity for distortion, leaving an intact semantic memory without associated illusory details.
- The third prediction is that whether a flat or graded RA curve is observed in rodent studies will depend on whether contextual information was repeatedly presented to the animal. If contextual information was presented more than once, some amount of this context will become semanticized, and thus hippocampal damage will lead to a flat RA gradient. However, if contextual information was presented only once, there isn’t an opportunity for semantic information to build up, and hippocampus damage will lead to a graded RA. Controlled experiments with amnesic patients or lesioned animals can test this prediction. It is interesting to note here that flat or graded RA curves can be obtained also with cortical lesions. Cho and Kesner (1996) showed that a flat RA gradient could be obtained with parietal cortical lesions while a temporally graded RA could be obtained with entorhinal cortical lesions in a spatial discrimination task. Thus, the location of the lesion, as well as the degree of contextual repetition are both hypothesized to influence the temporal gradient of RA.
- The fourth prediction is that the fidelity of mnemonic representations should change with repeated reactivations in the hippocampus and the neocortex. The degree of overlap in representations from exposure to exposure should predict how strong the memory is in a subsequent test (i.e., should generate a generalizable representation), while the degree of stochasticity should predict how contextual the memory is. Furthermore, given the hippocampus’ powerful capacity for orthogonalizing inputs, it may be the case that repetitions of identical study items may nonetheless induce competition as a result of varying external and internal contextual elements. This prediction can be tested using multivariate pattern classification techniques such as representational similarity analyses (Kriegeskorte et al., 2008) applied to fMRI or neurophysiological recording data.
- The fifth prediction is that there will be evidence for interference in the neocortex and not just the hippocampus, however, the timescale for neocortical trace interference will be much slower than the hippocampus and the mechanism will be more dependent on competitive inhibition via LTD-like mechanisms rather than pattern separation. This prediction may prove especially difficult to assess, though neurophysiological recordings and gene expression assays in animal models, or post-exposure representational similarity analyses via fMRI in humans may provide some insight. It is important to note here that LTD mechanisms are not hypothesized to be limited to the neocortex or to competitive inhibition *per se*. There is strong evidence that these mechanisms are also necessary for the acquisition of new memories in the hippocampus (Kemp and Manahan-Vaughan, 2004). Thus, this is likely a much more general mechanism, which could serve several purposes for our memory system.

## Conclusion

The role of the hippocampus in retrieval has been subject to much debate. The two schools of thought on the topic, SMSC and MTT, have had divergent predictions and so far, it is still not clear which model best describes the empirical data. We propose an alternative model in the form of a continuum and hypothesize that the role of the hippocampus during retrieval is recontextualization of memories along this continuum. This process, in turn, facilitates competition and trace interference in the cortex such that that consolidated memory traces become semantic. Our model explains much of the current data and provides fodder for future research in the form of testable empirical predictions. It may prove helpful as we shift our focus from categorical assignments such as “episodic” and move toward a more computationally grounded, cross-species compatible, continuum approach to declarative memory.

## Conflict of Interest Statement

The authors declare that the research was conducted in the absence of any commercial or financial relationships that could be construed as a potential conflict of interest.
